# Mechanism of Mepiquat Chloride Regulating Soybean Response to Drought Stress Revealed by Proteomics

**DOI:** 10.3390/plants12102037

**Published:** 2023-05-19

**Authors:** Shoukun Dong, Xin Wang, Xiaomei Li, Yumei Tian, Xinyu Zhou, Zhipeng Qu, Xiyue Wang, Lijun Liu

**Affiliations:** 1 Agricultural College, Northeast Agricultural University, Harbin 150030, China; 2 Heilongjiang Agricultural Engineering Vocational College, Harbin 150088, China

**Keywords:** soybean, growth regulator, osmotic stress, proteomics, mepiquat chloride

## Abstract

Soybeans are the main sources of oil and protein for most of the global population. As the population grows, so does the demand for soybeans. However, drought is a major factor that limits soybean production. Regulating soybean response to drought stress using mepiquat chloride (MC) is a feasible method; however, its mechanism is still unclear. This study used PEG-6000 to simulate drought stress and quantitative proteomic techniques to reveal changes in Heinong44 (HN44) and Heinong65 (HN65) subjected to drought following the application of 100 mg/L of MC. The results showed that SOD in HN44 did not change significantly but decreased by 22.61% in HN65 after MC pretreatment, and MDA content decreased by 22.75% and 21.54% in HN44 and HN65, respectively. Furthermore, MC improved the GSH–ASA cycle and simultaneously promoted the Calvin cycle process to enable the plant to maintain a certain carbon assimilation rate under osmotic stress. In addition, MC upregulated some proteins during gluconeogenesis and starch metabolism and increased soluble sugar content by 8.41% in HN44. MC also reduced ribosomal protein abundance, affecting translation and amino acid metabolism. In summary, MC improved GSH–ASA cycle and Calvin cycle under stress to alleviate oxidative damage and maintain crop growth. Our study is the first to report the mechanism of MC regulation in soybean under osmotic stress, providing new insights for the rational application of MC in soybean.

## 1. Introduction

Soybean is an important oil and economic crop, providing humans with rich oil and plant protein and playing a vital role in the human diet and industrial production [1,2]. Unfortunately, the current soybean production worldwide still cannot meet demand. One of the important reasons is that soybean is vulnerable to various abiotic stresses in the soybean growth process, such as drought, high temperatures, and floods [3,4]. Drought is the most widespread and common form of abiotic stress globally, and the annual yield loss caused by drought is approximately 25–50% [5]. Global warming and climate change are likely to increase this impact [6,7]. Drought decreases the amount of water available for plants from the soil, reduces stomatal closure to slow transpiration and a loss of water [8], followed by a decrease in photosynthesis and the carbon dioxide fixation rate, leading to slow plant growth, and a decrease in plant height, leaf area, and other morphological indicators [9]. Simultaneously, when the light energy absorbed by plants exceeds the energy plants need to fix carbon dioxide, reactive oxygen species (ROS) will be produced in photosystem I and photosystem II. The process of photorespiration induced via osmotic stress also leads to the accumulation of H_2_O_2_ in the peroxisome [10,11,12], an imbalance in intracellular ROS metabolism, resulting in damage to molecules such as nucleic acid and membrane lipids, and even plant death. Therefore, the ability to scavenge excess ROS in stress is crucial. Researchers and agricultural growers have attempted to use plant growth regulators to cope with drought stress.

The use of growth regulators considerably helps the planting industry, and various regulators play roles in different aspects of crop growth. Mepiquat chloride (MC) is an environmentally friendly, low-cost growth regulator that does not easily remain in crops. It is widely used in cotton production and can restrain the overgrowth of crops, adjust plant canopy structure, and increase the yield and quality of the cotton population [13]. MC also has some applications in other crops; however, the effects differ. MC in maize improves the process of plant lignin synthesis and enhances stem strength [14], whereas the use of MC in cassava promotes root formation, as well as increasing the starch content of root tuber and improving the quality of cassava [15]. However, studies on how MC regulates crop under stress are few, and related experiments all use MC as a seed-induced treatment agent [16,17]. Although this strategy is effective, MC is frequently used as a foliar spray in agricultural production.

In recent decades, the rapid development of omics technology has enabled researchers to explore plant changes at the subtle molecular level. In a previous study, we used transcriptome technology to analyze the effects of MC on soybean growth [18]. We found that the signal transduction of genes related to cell wall synthesis and hormones such as gibberellin and brassinolide was inhibited, with simultaneous changes in the secondary metabolic processes. This gave rise to a new question: how does MC regulate the drought resistance of soybean? In this study, PEG-6000 was used to simulate drought stress and proteomics was used to analyze the regulatory effect of MC on the soybean response to stress, which provided molecular insights into the MC regulation of soybean stress resistance. This study supplements studies on the effect of MC on the growth of soybeans, analyzes the mechanism regulating plant stress resistance, and provides a reference for the rational application of MC.

## 2. Results

### 2.1. Physiological and Biochemical Response in Soybean after MC Pretreatment

**Figure 1:**
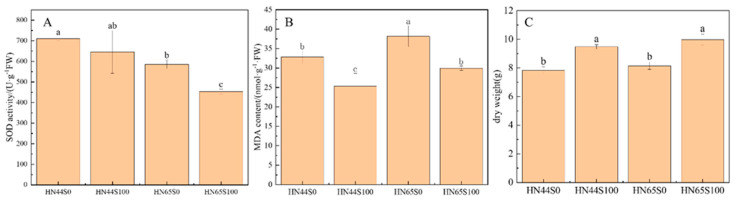
SOD activity (**A**), MDA content (**B**) and dry weight (**C**) in drought and MC treatment groups in HN44 and HN65 (S0: drought treatment group; S100: MC treatment group). Different letters in treatments indicate significant differences according to Duncan’s single factor variance test at the 5% level.

SOD and MDA can reflect the damage degree of crops under stress, and the higher their content, the more serious the damage to the plant. As shown in Figure 1A, after pretreatment with MC, the activity of SOD in HN44 did not change significantly, but decreased by 22.61% in HN65. In addition, the activity of SOD in the HN65 drought treatment group was lower than that of HN44, which proved that the response of the two varieties to drought was different. MDA was used to measure the damage degree of plants attacked by ROS, as shown in Figure 1B. HN44 and HN65 showed a decrease in MDA content after MC pretreatment, which proved that MC alleviated the damage of the membrane lipid caused by ROS. The significant changes in these two indicators proved that MC treatment can regulate the response of soybean to drought. In addition, we measured the dry weight of the plant, which was used to reflect the growth status of the crop, as shown in Figure 1C; the dry weight of both cultivars increased significantly after MC treatment, increasing by 21.23% in HN44 and by 22.45% in HN65, which indicated the positive effect of MC on soybean growth under drought.

### 2.2. Differential Accumulation Proteins of Drought Stress after MC Pretreatment

**Figure 2:**
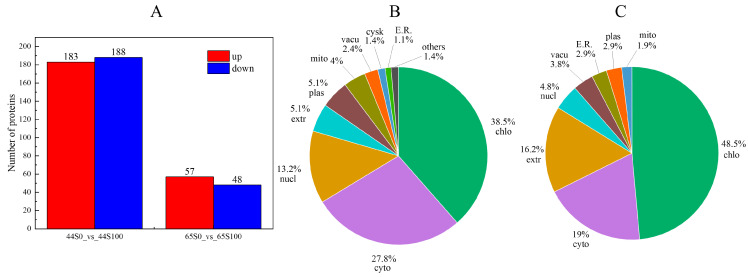
(**A**) The number of differential proteins between the MC and drought treatment group. Red represents the protein upregulated by MC, and blue represents the downregulated proteins. (**B**) Subcellular localization of DAPs in HN44. (**C**) Subcellular localization of DAPs in HN65 (S0: drought treatment group; S100: MC treatment group).

Proteomic analysis was conducted to explore the effect of MC pretreatment on soybean response to drought stress, and 9155 proteins were identified. Differential proteins were screened in the drought (S0) and MC (S100) treatment groups. Based on the criteria of *p* < 0.05 and FC > 1.2 or < 0.83, 371 proteins (183 upregulated and 188 downregulated) and 105 proteins (57 upregulated and 48 downregulated) were identified as differentially accumulated proteins (DAPs) in HN44 and HN65, respectively (Figure 2A). Subcellular localization analysis of differentially accumulated proteins in two varieties showed that DAPs in HN44 were mainly located in chloroplasts and the cytoplasm, accounting for more than 60%. More differentially expressed proteins were observed in the nucleus, with fewer proteins being observed in the cytoskeleton and endoplasmic reticulum (Figure 2B). Most DAPs in HN65 were located in the chloroplasts and cytoplasm, and many differential proteins were observed in the extracellular region (Figure 2C).

### 2.3. GO Enrichment Analysis

**Figure 3:**
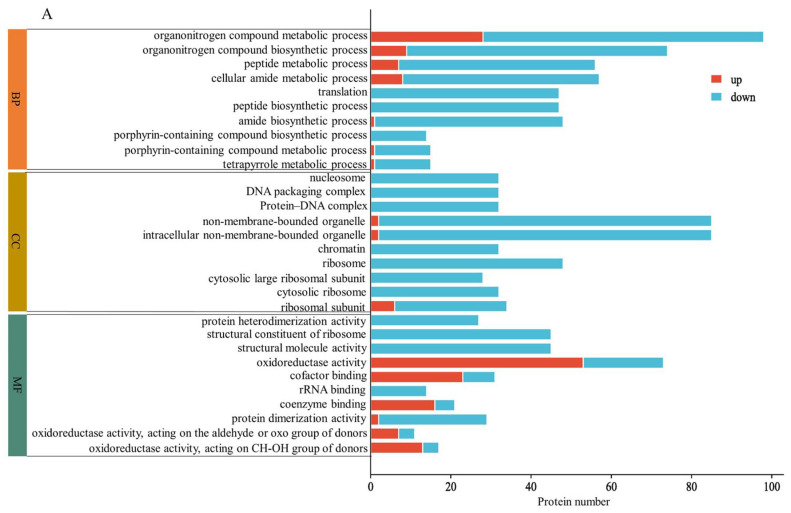

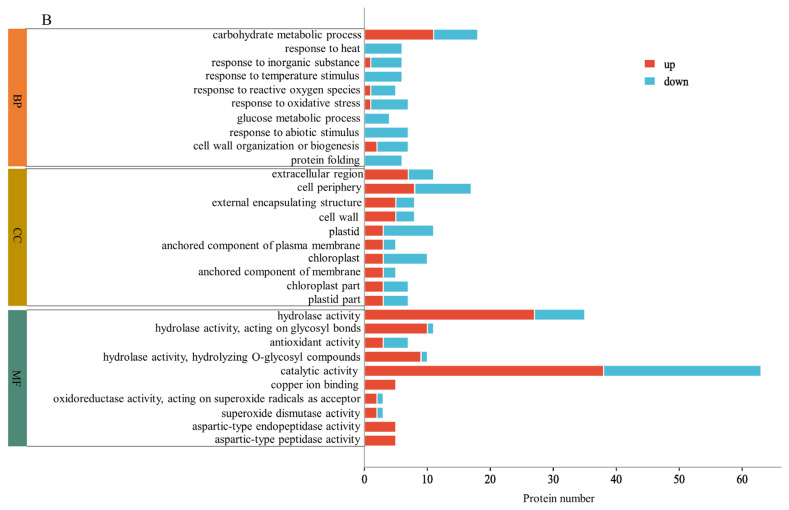
GO enrichment (*p* < 0.05) for DAPs of MC treatment on different varieties, HN44 (**A**) and HN65 (**B**).

GO analysis revealed the enrichment of DAPs in biological processes, cellular components, and molecular functions induced via MC pretreatment. Figure 3A shows the enrichment results for DAPs in HN44-S0 and HN44-S100. Regarding biological processes, the decomposition and synthesis of organic nitrogen compounds, peptide metabolism and synthesis, translation, amide metabolism and synthesis, and porphyrin metabolism and synthesis were significantly enriched, and all proteins were downregulated during translation and peptide synthesis. Regarding cellular components, the DAPs were enriched in nucleosomes, DNA–protein complexes, and chromatin, and all proteins related to the ribosome (cytoplasmic ribosome and ribosomal subunit) were downregulated. For molecular function, the number of upregulated proteins was largest in terms of oxidoreductase activity, cofactor binding and coenzyme binding. In addition, DAPs were significantly enriched in protein heterodimerization activity and in the structural constituent of the ribosome; however, all related proteins were downregulated. Figure 3B shows the enrichment results for DAPs in HN65-S0 and HN65-S100. In biological processes, DAPs were enriched in carbohydrate metabolism and downregulated in several terms related to the stress response, confirming MC’s regulation of stress tolerance. Additionally, cell wall organization or biogenesis was significantly enriched. Among the cell components, DAPs were enriched in the cell wall, anchored component of the plasma membrane, chloroplasts, and plastids. In terms of molecular functions, the main enriched ones were hydrolase activity, antioxidant activity, superoxide dismutase activity, and aspartic-type endopeptidase activity. The GO-enriched items differed in the two varieties, proving that the response of MC pretreatment to drought varied with the varieties. However, the changes in photosynthesis, amino acid or peptide metabolism, carbohydrate metabolism, and nitrogen metabolism confirmed the regulatory effect of MC on plant growth under drought. Simultaneously, the enrichment of redox-related enzyme activity proved its effect on the stress resistance of soybean.

### 2.4. KEGG Enrichment Analysis

**Figure 4:**
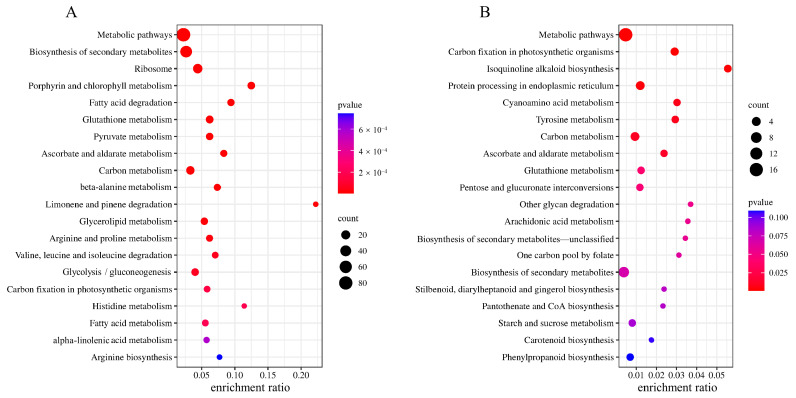
KEGG enrichment scatter plot. The larger the dot, the higher the number of differential proteins enriched by the pathway. The redder the color of the dots, the more significant the enrichment. (**A**) HN44 group and (**B**) HN65 group.

The DAPs of HN44 and HN65 were analyzed using KEGG enrichment analysis. The first 20 KEGG pathways are shown in Figure 4. In HN44, DAPs were enriched in ribosomes, porphyrin, chlorophyll metabolism, fatty acid degradation, glycerol ester metabolism, and ascorbate and aldarate metabolism. The enrichment pathways in HN65 differed, and the main DAPs were enriched in carbon fixation, cyanoamino acid metabolism, tyrosine metabolism, and the isoquinoline alkaloid synthesis pathway in photosynthesis. Based on the enrichment pathways, HN44 showed extensive changes in amino acids, fatty acids, and other substances, whereas HN65 showed active amino acids and secondary metabolism. In addition, KEGG enrichment analysis showed that glutathione metabolism (ko00480), ascorbate acid and aldarate metabolism (ko00053), carbon metabolism (ko01200), and carbon fixation in photosynthesis (ko00710) were enriched in both HN44 and HN65. It is suggested that the above pathway may be a key step for MC to regulate the response of soybeans to drought stress.

### 2.5. MC Pretreatment Improved the Glutathione–Ascorbic Acid Cycle and Alleviated Oxidative Damage

**Figure 5:**
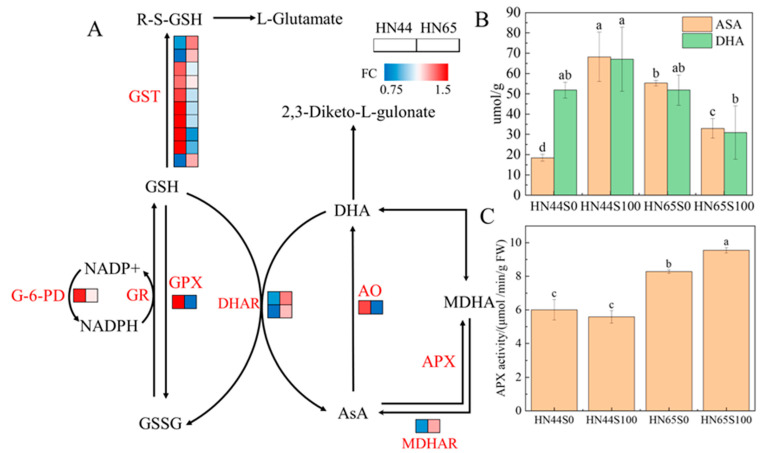
(**A**) ASA–GSH cycle under MC treatment. (**B**) ASA and DHA contents under drought and MC treatments. (**C**) APX activity under drought and MC treatments. Multiple comparisons were performed between four treatments of the same index (S0: drought treatment group; S100: MC treatment group). Different letters in treatments indicate significant differences according to Duncan’s single factor variance test at the 5% level.

Glutathione (GSH) and ascorbic acid (ASA) are important non-enzymatic antioxidants in plants that play important roles in the response of plants to biotic and abiotic stresses. After plants were pretreated with MC, some proteins of the two varieties were enriched in glutathione metabolism (ko00480) and ascorbate and aldarate metabolism (ko00053). However, the performances of HN44 and HN65 were not identical, as shown in Figure 5A. After pretreatment with MC, the abundance of glutathione-S-transferase (GST) changed significantly, in which two dehydroascorbate reductase (DHAR)-type proteins and one lambda-type GST protein were specifically increased in HN65, whereas the proteins specifically upregulated in HN44 belonged to tau-type GST and phi-type GST. DHAR and lambda-type GST are thiotransferases that are similar to peroxidases. Under chemical and oxidative stress, DHAR and lambda in Arabidopsis thaliana root cells are upregulated, indicating that these two types of GST are involved in cellular oxidative stress [19]. Tau-type GST can bind to various harmful chemical herbicides to protect cells from toxicity. Simultaneously, the transcription level of the tau-type GST increases significantly under drought induction [20], indicating that the tau-type GST also plays a role in oxidative stress.

Oxidative stress induced by ROS is an important factor in plant cell damage after drought stress. Therefore, plants activate various antioxidant pathways under stress conditions. After MC pre-treatment, the activity of HN44 glutathione reductase (GR) increased, promoting the synthesis of reduced glutathione. Glutathione can bind to free radicals to protect protein sulfides, which regulate important biological activities and prevent their inactivation. Simultaneously, the expression of glutathione peroxidase (GPX), which reduces hydrogen peroxide by converting reduced glutathione into oxidized glutathione, is upregulated. The upregulation of GR and GPX indicated that MC accelerated the conversion of reduced glutathione and oxidized glutathione to deal with the free radicals produced via drought stress and prevented the damage of other proteins in the cells; however, this process was not observed in HN65. Dehydroascorbate reductase (DHAR) and ascorbate oxidase (AO) were specifically upregulated in HN65. These two enzymes catalyze the reduction of DHA to ASA and the conversion of ASA into DHA, respectively. In addition, ASA can be converted into mono-dehydroascorbic acid (MDHA) by APX and then spontaneously into DHA via a non-enzymatic reaction. APX-related proteins were not observed in the proteomic data; however, we measured APX activity. The APX activity of HN44 after MC treatment was not significantly different from that under drought conditions, whereas the HN65APX activity was significantly upregulated by 15.34% after MC application (Figure 5C). MDHA was reconverted to ASA by monodehydroascorbate reductase (MDHAR), and the abundance of related proteins was upregulated in HN44 but not in HN65. In addition, the ASA and DHA contents were determined, as shown in Figure 5B. After MC pretreatment, the content of ASA in HN44 leaves increased by 269.96%, but the content of DHA did not change significantly. However, the content of ASA decreased significantly in HN65, but the change in DHA content was not significant. The changes in the two varieties were completely different owing to the different responses of the two varieties to drought.

In summary, the glutathione and ascorbic acid cycles were activated under drought stress after MC pretreatment, and higher levels of antioxidant enzymes and glutathione S-transferase played a protective role in drought stress compared with plants without MC spraying. Specifically, this pathway is one of the methods of MC pretreatment with which to enhance the drought tolerance of plants.

### 2.6. MC Pretreatment Affects Carbon Metabolism in Soybean

**Figure 6:**
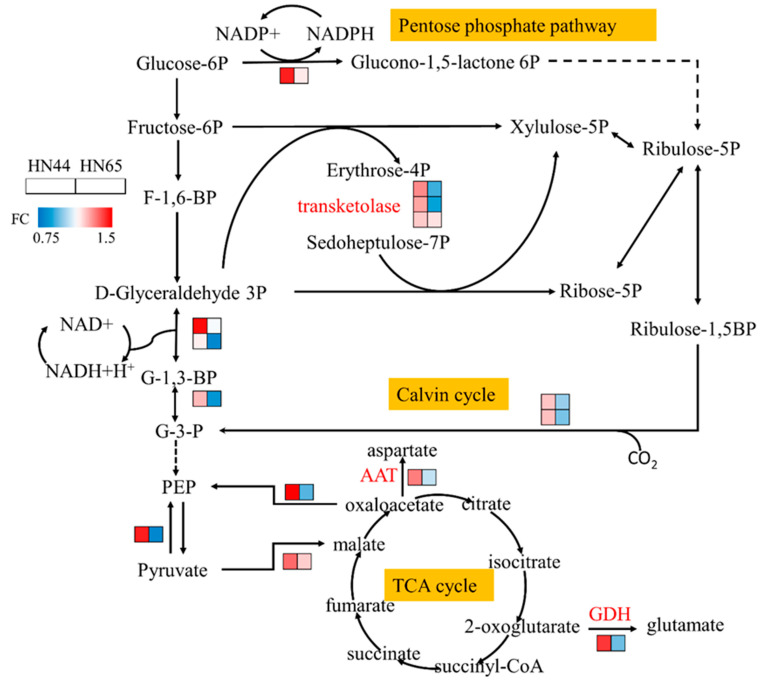
Carbon metabolism map under MC treatment.

As shown in Figure 6, the proteomic data showed that the abundance of glucose-6-phosphate1-dehydrogenase increased in HN44, which produces nicotinamide adenine dinucleotide phosphate (NADPH), one of the reactions that provide reducing power for the synthesis of other substances. The pentose phosphate pathway also starts from this step. After MC treatment, the abundance of transaldolase increased, and the contents of erythritose-4-phosphate, heptanone-7-phosphate, xylulose-5-phosphate, and other downstream substances increased, providing reaction precursors for the synthesis of nucleic acids, amino acids, and secondary metabolites. The protein abundance of glyceraldehyde-3-phosphatedehydrogenase (GAPD) and phosphoglycerate kinase (PGK) increased, and GAPD and PGK catalyzed the transformation from glycerol triphosphate into glyceraldehyde-3-phosphate, which simultaneously involved the dark reaction of photosynthesis and glycolysis. However, no significant change in the activity of other enzymes in glycolysis was observed, and the protein abundance of most enzymes changed during the dark reaction in this experiment. Therefore, we believe that the changes in GAPD and PGK activity under the MC treatment were mainly involved in the dark reaction process of photosynthesis. MC pretreatment improved the Calvin cycle during photosynthesis and enabled soybeans to maintain a certain carbon assimilation rate under drought stress.

**Figure 7:**
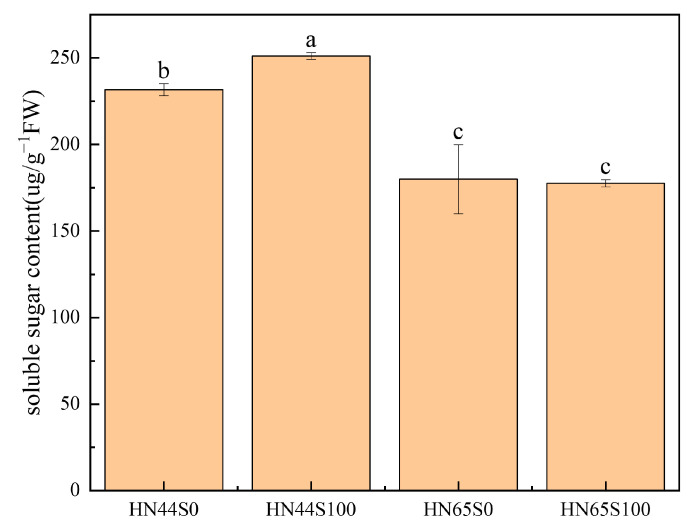
Soluble sugar content under MC and drought treatments (S0: drought treatment group; S100: MC treatment group). Different letters in treatments indicate significant differences according to Duncan’s single factor variance test at the 5% level.

The abundances of pyruvate, phosphate dikinase, and phosphoenolpyruvate carboxykinase increased, consuming ATP to convert pyruvate into phosphoenolpyruvate and participating in the process of gluconeogenesis. The glycolysis/gluconeogenesis pathway in plants has been repeatedly confirmed to be involved in the drought response [21,22], which may play a major role in accumulating sugars and protecting cells. We determined the soluble sugar content in the leaves of the drought and MC treatment groups, as shown in Figure 7, and found that the soluble sugar content in the leaves increased by 8.41% after MC pretreatment. Combined with the proteomic data, MC was shown to induce gluconeogenesis.

No evident change in protein abundance was observed in the tricarboxylic acid cycle; however, the conversions of aspartate aminotransferase from oxaloacetic acid into aspartic acid and of glutamate dehydrogenase protein from α-ketoglutaric acid into glutamic acid were enriched. Changes in aspartic acid and glutamate contents affect the metabolic processes of arginine, phenylalanine, and other amino acids. Notably, most of the proteins involved in the above-mentioned carbon metabolic pathways showed a downward trend in HN65. This indicates that the changes in physiological processes caused by MC pretreatment vary among different varieties.

### 2.7. The Unique Response of the Two Varieties Reveals a Protein–Protein Interaction Network

**Figure 8:**
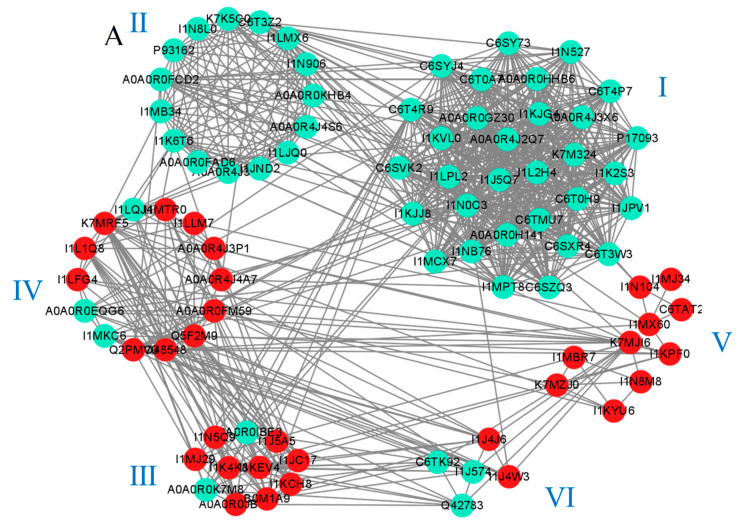

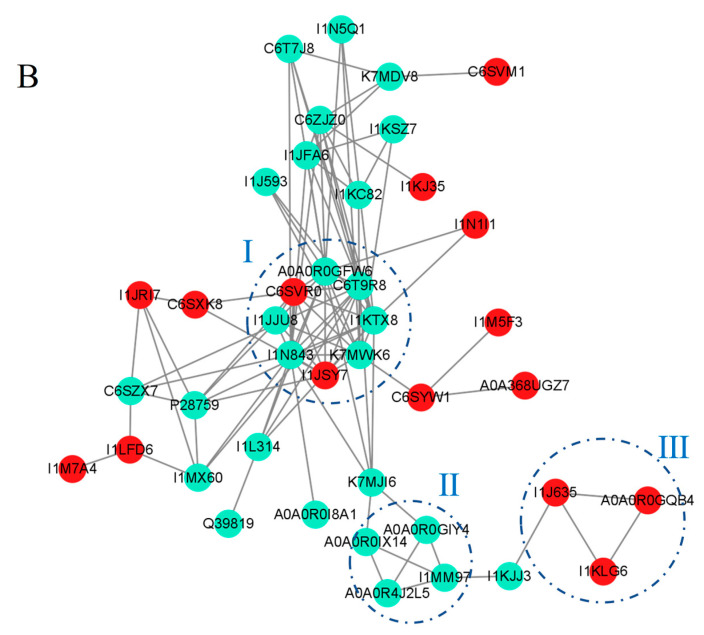
Protein–protein interaction networks of DAPs in HN44S0/HN44S100 (**A**) and HN65S0/HN65S100 (**B**) comparisons. The color of the circles indicates the difference in protein abundance. The upregulated proteins are marked in red, and the downregulated proteins are marked in cyan.

A protein–protein interaction network was constructed using a STRING database to understand the interactions between proteins. In HN44, a total of 246 proteins were mapped to an interaction network. We obtained six individually separated clusters using the MCODE plug-in in Cytoscape to screen all the proteins, as shown in Figure 8A. Cluster I contained 31 proteins, which were all annotated as ribosome-related proteins and were downregulated after MC pretreatment. In addition, the CytoHubba plug-in was used to predict that 20 may be key proteins in the response to MC preconditioning and drought stress (Figure S1), which were all enriched in the KO03010 pathway, further proving the important role of ribosomes in the drought response to MC application. As key macromolecules in the translation process, a decrease in ribosomal protein abundance indicates that plant protein synthesis is negatively affected. Cluster II contained 15 proteins related to porphyrin and chlorophyll metabolism. The expression of these 15 proteins was downregulated. Drought stress adversely affects plant chlorophyll content. However, MC had no positive effect on chlorophyll content. There were 11 proteins in Cluster III centered around I1KEV4 and I1K4K8. I1KEV4 and I1K4K8 are acyl-CoA oxidase proteins that catalyze the dehydrogenation of saturated fatty acids, and the abundance of these two proteins was upregulated, indicating that MC pretreatment can withstand drought stress via regulating the proportion of intracellular fatty acids. Cluster IV comprised 14 proteins. Annotation results showed that these proteins were closely related to amino acid metabolism. Except for the downregulation of I1LQJ4, I1MKC6, and A0A0R0EQG6, the remaining 11 proteins were upregulated. This is consistent with the changing trend of Cluster I, which we believe is the result of the coordination of energy and reaction substrate distributions in plants. The translation process consumes a large amount of energy to connect amino acids to polypeptide chains. In KEGG enrichment analysis, MC pretreatment caused changes in the metabolism of many kinds of amino acids in HN44 (Figure 3), which indicated that MC pretreatment reduced the distribution of amino acids and energy in the translation process via reducing ribosomal protein abundance, thus activating some secondary metabolic and amino acid metabolism processes. Cluster V contained 10 proteins related to the glutathione cycle and starch–sucrose metabolism. The abundances of alpha-glucan phosphorylase (I1KYU6) and sucrose synthase (K7MZJ0), which catalyze the conversion of d-phosphate glucose into UDPG and the synthesis of sucrose from UDPG and fructose, respectively, were upregulated. In addition, the abundance of 4-alpha-glucanotransferase (I1N8M8) and alpha-1,4glucanphosphorylase (I1KYU6), which catalyze the hydrolysis of maltose and starch into glucose monomers, was also upregulated, possibly providing important reaction substrates for other metabolic processes. Simultaneously, four glutathione metabolism-related proteins were also upregulated.

The changes in HN65 and HN44 caused by MC pretreatment were different. Fifty proteins were mapped to a PPI network. As shown in Figure 8B, three clusters were obtained using the MCODE plug-in. Two plastocyanin proteins in Cluster I were upregulated, and the rest were downregulated, including the chlorophyll ab-binding protein, glyceraldehyde 3-phosphate dehydrogenase, and the RuBisco protein’s small subunit. Cluster II contained downregulated carotenoid cleavage dioxygenase4 (A0A0R4J2L5), a key protein for the synthesis of abscisic acid, which proves that the process of ABA synthesis is inhibited. The ABA content in plants can change dozens of times under drought conditions [23], and is considered one of the responses to drought stress. ABA binds to the PYR/PYL receptor and activates downstream PP2C and SNRK2 family protein kinases, resulting in changes in stomatal conductance and proline and chlorophyll content. Simultaneously, ABA antagonizes auxin and gibberellin to compete for the reaction precursor of chlorophyll synthesis and inhibit plant growth; therefore, the content of ABA is strictly regulated, and MC treatment leads to a decrease in ABA synthesis. This shows that plants no longer need an excessive ABA content; that is, the drought damage to plants is reduced. Cluster III contains two phenol oxidase proteins (I1KLG6 and A0A0R0GQB4), and their abundance is upregulated, indicating that MC pretreatment leads to the activation of phenol oxidase-catalyzed secondary metabolism and tyrosine–phenylalanine conversion, which is considered an adaptive response of plants to cope with changes in the external environment.

### 2.8. Validation of Proteomic Analysis

**Figure 9:**
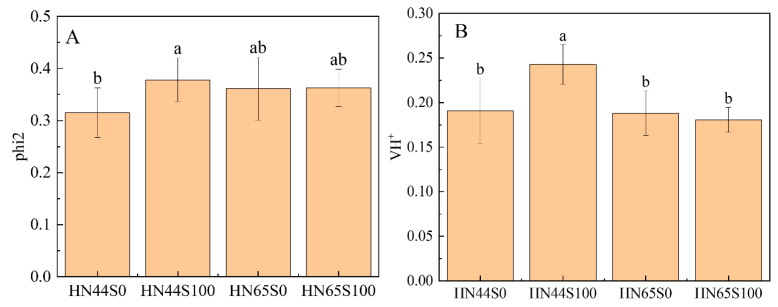
Photosynthetic parameters of two varieties under MC treatment and drought treatment. (**A**) Actual photosynthetic efficiency (phi2) and (**B**) proton kinetic potential (VH+). Different letters in treatments indicate significant differences according to Duncan’s single factor variance test at the 5% level.

To validate the proteomic data, some genes were determined via qRT-PCR. The proteins encoded by these genes are involved in amino acid metabolism, isoflavone synthesis, carbon metabolism, and glycolysis/gluconeogenesis. As is shown in Figure S5. The change trend of the relative expression level of the genes was basically consistent with the change trend of its encoded proteins. Only the change in phosphoenolpyruvate carboxykinase (ATP) 1 encoded by LOC100791015 was inconsistent in HN65. The difference may be caused by post-transcriptional regulation and modification, and this process has variety specificity. Generally, the change trend in the expression level of most genes is consistent with that of proteins, which proves the reliability of the proteomic data. In addition, biochemical indicators were used to assist in verifying the reliability of omics data [24], as shown in Figure 9A. The phi2 reflects the ability of light energy to be converted into ATP and NADPH and finally synthesize sugar, which is affected by the adverse environment. The phi2 index was significantly upregulated after MC treatment in HN44, but not changed in HN65, which was similarly to the trend shown in proteomics (proteins related to the carbon assimilation process were upregulated in HN44, but not significantly changed or downregulated in HN65). Figure 9B shows the change in leaf proton dynamic potential (VH+), which reflects the ability of ATP synthase to convert ADP into ATP. MC pretreatment led to the VH+ being increased in HN44, while it led to no significant change in HN65. According to the proteomic data, the protein content of ATP synthase increased in HN44, which was consistent with the change in photosynthetic parameters.

## 3. Discussion

Drought is one of the most frequently occuring and serious natural disasters affecting crop growth. Drought adversely affects the growth or yield of crops during the entire growth period, and the water deficit in the seedling stage will lead to slow plant growth and weak seedlings [25]. Drought stress at the seedling stage decreases plant height and leaf area [26] and significantly inhibits dry weight accumulation [27]. Although there has been evidence that temporary drought and water recovery are beneficial to plant nutrient absorption and growth [28], owing to climate uncertainty in production, the effects of drought cannot be underestimated. The production of ROS is the main cause of plant cell damage and even death under stress [29]. Under drought conditions, an imbalance exists between ROS production and metabolism, and one of the products of reactive oxygen species attacking membrane lipids is MDA, which is commonly used to measure the degree of plant damage [30]. In this experiment, the MDA content decreased after using MC, which is favorable evidence that supports the use of MC for alleviating drought. There are two possible reasons for this decrease in MDA levels: an increase in the scavenging ability of ROS and a decrease in ROS production. The GSH–ASA cycle is affected by MC pretreatment, in which glutathione and ascorbic acid play important roles in cell defense against oxidative damage. In this cycle, enzymes, such as GST, perform the function of scavenging ROS. GST8 in the GST family responds to rapid dehydration. Drought-induced changes in GST8 transcriptional abundance were also observed in aba1-1-deficient mutants [31]. Changes in the protein abundance of the GST family were observed in two varieties in this experiment, but their categories were different. In addition to GST, GPX and APX are also reported to be antioxidant enzymes [32], activated via ROS signals, which catalyze the transformation of the oxidation and reduction forms of GSH and ASA, while reducing hydrogen peroxide to water. Notably, they were not simultaneously activated in the two experimental varieties and specific changes in GPX and APX were observed in HN44 and HN65, respectively.

Photosynthesis is an important biological process regulated via drought and MC. Plants under drought stress show a decrease in stomatal conductance and chlorophyll content; therefore, light capture, photosynthetic electron transport, and the Calvin cycle are affected [33]. However, the effect of MC application on photosynthesis is different. Early experiments found that MC treatment could improve the light energy use efficiency of cotton and promote the stomatal opening and CO_2_ exchange rate of cotton [34], which was caused by an increase in leaf area; however, the stomatal opening implies more water loss from transpiration, which possibly impacts plants under drought conditions, which remains to be discussed. It was subsequently reported that the photosynthesis and final yield of cotton decreased after the use of MC [35], and it was also reported that MC decreased the activity of Rubisco, thus reducing the net photosynthetic rate (Pn) of leaves [36]. Completely different conclusions show that the regulation of photosynthesis via MC is more complex, and that the effect of MC on photosynthesis varies with plant species. A decrease in the transpiration rate and stomatal conductance was observed in the woody plant eucalyptus [37]. In this experiment, proteins regulating photosynthesis showed different trends even in the same species. In HN44, the proteins related to ferredoxin (Fd), photosystem I, and photosystem II in the photosynthetic electron transport chain were downregulated, but the ATP synthase protein was upregulated, whereas only plastocyanin was upregulated in HN65. Photosynthesis provides ATP, [H], and other substances for plant growth, and consumes ATP to fix carbon dioxide, which is the basis of plant growth. MC treatment improves the process of carbon assimilation to a certain extent; however, MC cannot completely eliminate the negative effects of drought stress on photosynthesis and chlorophyll synthesis-related proteins are still decreased, which we believe is a state of equilibrium after adaptation. Because the proteins in the electron transport chain are inhibited, a high chlorophyll content fixes a large amount of light energy, and excess energy leads to the production of ROS and damage to cells and chloroplasts [38]. Generally, MC alleviates the inhibition of carbon assimilation caused by drought, allowing plants to maintain the process of carbon assimilation to ensure growth (this is evidenced by a positive trend in plant dry matter accumulation). The cells are protected from strong light by maintaining a low chlorophyll content and a low electron transfer rate.

According to Herms [39], plants cannot move their positions to avoid adverse growth factors, and in the long process of evolution, plants can adjust their growth state to adapt to the environment to ensure their survival under adversity. Under biotic or abiotic stress, plants regulate their metabolism and synthesize more secondary metabolites, such as anthocyanins, flavonoids, and terpenes [40]. Most of the synthesis of secondary metabolites uses primary metabolites (such as various amino acids) as reaction substrates. Proteomic data show that ribosomal proteins decrease and the translation process is inhibited, indicating that amino acids may be transported to other biological processes to perform their biological functions, such as protecting cells in the form of free amino acids [41], or as a substrate to synthesize alkaloids and flavonoids [42]. Consistently with our results, Cui et al. found that plants appear to need many amino acids under stress conditions; nitrogen uptake and amino acid synthesis were significantly affected under drought stress, and the expression of related genes was upregulated [43]. Improving nitrogen fertilizer application could alleviate drought injury [44], and some studies showed that protein synthesis was inhibited under salt stress [45]. Increasing the synthesis of amino acids and reducing the conversion of amino acids to peptides is an alternative way for plants to overcome stress, and MC pretreatment also plays a similar role. In addition to extensive changes in amino acids, sugars in cells are also affected by MC. Under drought stress, plants usually accumulate soluble sugars to maintain intracellular osmotic potential and prevent a large amount of cell water loss [46]. MC treatment significantly increased the content of soluble sugars in HN44, which was consistent with the gluconeogenesis process and the upregulation of proteins related to starch decomposition observed in proteomics. In summary, MC regulates the composition and content of amino acids, as well as the transformation of sugar content, maintains high cellular osmotic potential, and provides reaction substrates for other life activities (such as secondary metabolism) to withstand drought.

## 4. Materials and Methods

### 4.1. Experimental Materials and Treatment

Heinong 44 (HN44) and Heinong 65 (HN65), the two main soybean varieties in Northeast China, were planted with sand in plastic buckets of a diameter of 25 cm and height of 30 cm, leaving three seedlings in each pot. Soybean seeds were obtained from the Heilongjiang Academy of Agricultural Sciences.

The experiment established drought (S0) and MC (S100) treatment groups. Soybeans in both groups were irrigated with distilled water before euphylla expansion and with 500 mL of the Hoagland nutrient solution daily during the period between euphylla expansion and seedling growth (V3) [47]. When the soybeans grew to the V3 stage, 100 mg/L of the MC solution was evenly sprayed on the leaves of the MC treatment group (S100), whereas the drought treatment group (S0) was not sprayed with this. After three days, both groups were irrigated with 15% (*m*/*v*) PEG-6000 for the Hoagland nutrient solution. PEG-6000 induces osmotic stress, and was used to simulate drought in this experiment. PEG irrigation lasted for four days and samples were collected after four days. Each treatment was repeated three times. Each indicator was repeated three times to ensure the accuracy of the data.

### 4.2. The Proteomics Determination Method

Based on the previous method [48], 1.5% SDS/100 mMTris-CL was added to soybean leaves, centrifuged, and acetone precipitation was used to precipitate proteins, then adding 8 MUrea/100 mMTris-CL was added to it. Then, using DTT, incubate at 37 °C for 1 h, add iodoacetamide for alkylation reaction in the dark. The standard curve was drawn with bovine serum albumin, and Bradford staining solution was prepared with Coomassie brilliant blue, ethanol and phosphoric acid, and then diluted with distilled water. Then, the staining solution was added to the samples, reacted for a few minutes, the absorbance was measured at 595 nm for quantitative identification of the protein. Then, 50 ug samples were used for SDS-PAGE detection. Tris-CL solution (100 mM) was added to the alkylated sample, trypsin was added at a ratio of 1:50, shaken at 37 °C, TFA was then added to stop digestion, and the supernatant was collected for Sep-PakC18 desalting and stored at −20 °C. TMT labeling was carried out according to the manufacturer’s instructions (ThermoScientific, Waltham, MA, USA), followed by Sep-PakC18 desalting, and HIGH-PH reverse chromatographic separation was used to separate the samples, and the samples were kept at −80 °C for later use.

### 4.3. Mass Spectrometry Detection and Data Analysis

An Orbitrap Exploris 480 mass spectrometer (Thermo Fisher Scientific, Dreieich, Germany) coupled with an EASY-nLC 1200 liquid-phase liquid chromatography-mass spectrometry (LC-MS) system (Thermo Fisher Scientific, Waltham, MA, USA) was used for the collection. Two mobile phases (mobile phase A: 0.1% formic acid; mobile phase B: 0.1% formic acid and 80% ACN) were used to establish an analytical gradient. Raw MS data were analyzed using MaxQuant (V1.6.6). The UniPort Glycine Max 20190126.fasta(UP000008827) database was used to search for the sequence, and the mass spectrometry-matching tolerance was set to 20 ppm. The search results were screened via 1% FDR at the protein and peptide levels, the contaminated proteins and protein entries with only one modified peptide were removed, and the remaining information was used for subsequent analysis. According to the relative quantitative results of the protein, the fraction of the mean value of protein in each biological repetition was used as the fold change (FC value).

GO and KEGG database annotations were performed using BLAST alignment (blastp, evalue ≤ 1 × 10^−5^). Enrichment analysis was based on Fisher’s exact test and Benjamini–Hochberg correction (*p* < 0.05). Protein interaction analyses were performed using the StringDB protein interaction database and visualized using Cytoscape (version 3.9.1).

### 4.4. Determination of Physiological and Biochemical Indexes

The plants were killed for 30 min at 105 °C, dried at 65 °C for 72 h, and then the dry weight was determined.

The contents of ascorbic acid (ASA) and dehydroascorbic acid (DHA) were determined using a modified method [49]. Soybean leaves (0.1 g) were ground into a homogenate by adding a buffer solution. The supernatant, after centrifugation was added to TCA, anhydrous ethanol, phosphoric acid–ethanol, BP–ethanol, and FeCl_3_, during a reaction at 30 °C for 90 min to determine ASA. For DHA, DTT and disodium hydrogen phosphate were first added; DHA was reduced to ASA, and then determined according to the same ASA method, and the final differentials were DHA content.

SOD and APX activity were determined using a kit provided by Norminkoda (Wuhan, China).

Soluble sugar content was determined via the anthrone colorimetric method based on the Gurrieri [50].

Malondialdehyde (MDA) content was determined via the the thiobarbituric acid method in accordance with the method by Rashid et al. [51].

The photosynthetic parameters of the leaves were measured using the photosynthetic measuring instrument MultispeQV2 based on the PhotosynQ cloud platform (Huinuo Reide Technology Co., Ltd., Beijing, China).

### 4.5. Real-Time Quantitative Polymerase Chain Reaction Detection Method

Utilizing MonScript™ RTIII Allin-One Mix with dsDNase, reverse transcription of whole RNA into cDNA was performed. The primers used in qPCR are presented in Supplementary Table S1. The PCR process was performed as follows: first, the initial heat activation of PCR was performed at 90 °C for two minutes for one cycle. Secondly, denaturation was performed at 95 °C for 5 s, combined annealing/extension at 60 °C for 30 s, in 40 cycles. Finally, the melting curve was analyzed. To determine the relative expression of the genes, the internal reference gene ACT11 was used, and the 2^−ΔΔCt^ method was applied. The instruments and models used in the test are as follows: PCR amplifier (846-x-070-301, Biometra TONE, Göttingen, Germany) and Gel imager (Tanon 5200 Multi, Tanon, Shanghai, China).

### 4.6. Statistical Analysis

Statistical analyses were performed using IBM SPSS software (version 23.0; IBM Corporation, Armonk, NY, USA), and figures were produced using OriginPro 2021 software (OriginLab Corp., Northampton, MA, USA).

## 5. Conclusions

This experiment was conducted to study how MC regulates the response of soybean under drought. In summary, MC improved the GSH–ASA cycle, regulated the content of antioxidant enzymes and non-enzymatic antioxidants, and enhanced the defense ability of plants under stress. MC also improved the Calvin cycle, affected the carbon metabolism process, and enabled plants to maintain a certain carbon assimilation rate under stress. At the same time, MC affected the nitrogen metabolism process and provided substrates for other life activities (such as secondary metabolism). Our results provided a reference for the development of other growth regulators; that is, we should consider both the defense state and growth state of crops in order to achieve the desired stress-resistant effect.

## Data Availability

The raw data were uploaded to the iProX proteomics mass spectrometry data storage platform, number PXD040486 (http://proteomecentral.proteomexchange.org/cgi/GetDataset?ID=PXD040486 (accessed on 31 March 2023)).

## Supplementary Materials

The following supporting information can be downloaded at: https://www.mdpi.com/article/10.3390/plants12102037/s1, Figure S1: Top 20 proteins predicted by the Cytohubba plugin in HN44. Figure S2: Top 20 proteins predicted by the Cytohubba plugin in HN65. Figure S3: The photos of HN65. Figure S4: The photos of HN44. Figure S5: Results of qRT-PCR. Table S1: qRT-PCR primer information.
