# Supplementary material for: Mechanism of Mepiquat Chloride Regulating Soybean Response to Drought Stress Revealed by Proteomics
Source: Plants (Basel). 2023 May 19;12(10):2037. doi: 10.3390/plants12102037 (PMC10222127; doi:10.3390/plants12102037)
Supplement: Supplementary file 1 [file plants-12-02037-s001.zip › Supplementary Figure S5.pdf]

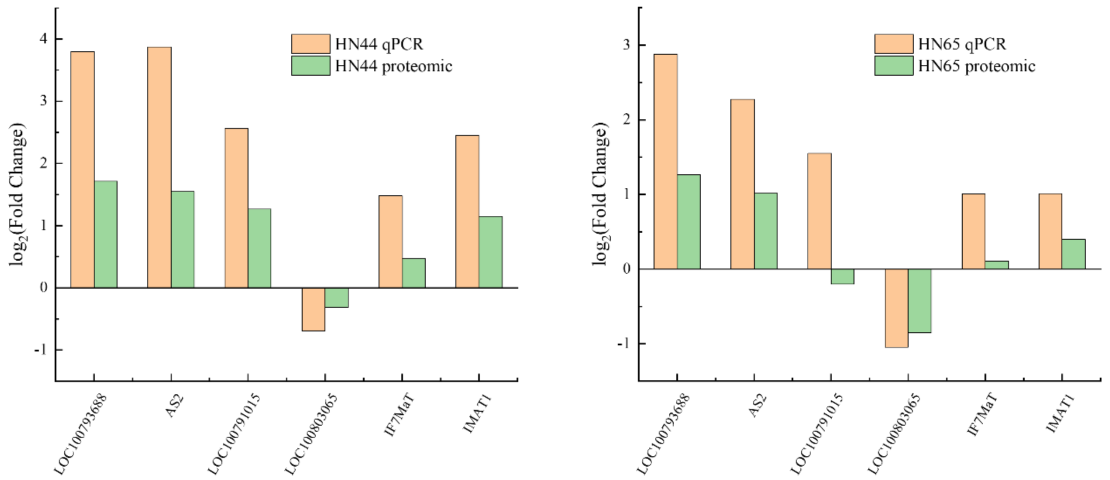

Supplemental Figure S5. The relative changes of genes and their encoded proteins in the two varieties, for each gene, bars represent the log2 values of fold changes on MC treatment group (S100) compared to that on drought treatment (S0).
