# Supplementary material for: Mechanism of Mepiquat Chloride Regulating Soybean Response to Drought Stress Revealed by Proteomics
Source: Plants (Basel). 2023 May 19;12(10):2037. doi: 10.3390/plants12102037 (PMC10222127; doi:10.3390/plants12102037)
Supplement: Supplementary file 1 [file plants-12-02037-s001.zip › Supplementary Figures S1 and S2.pdf]

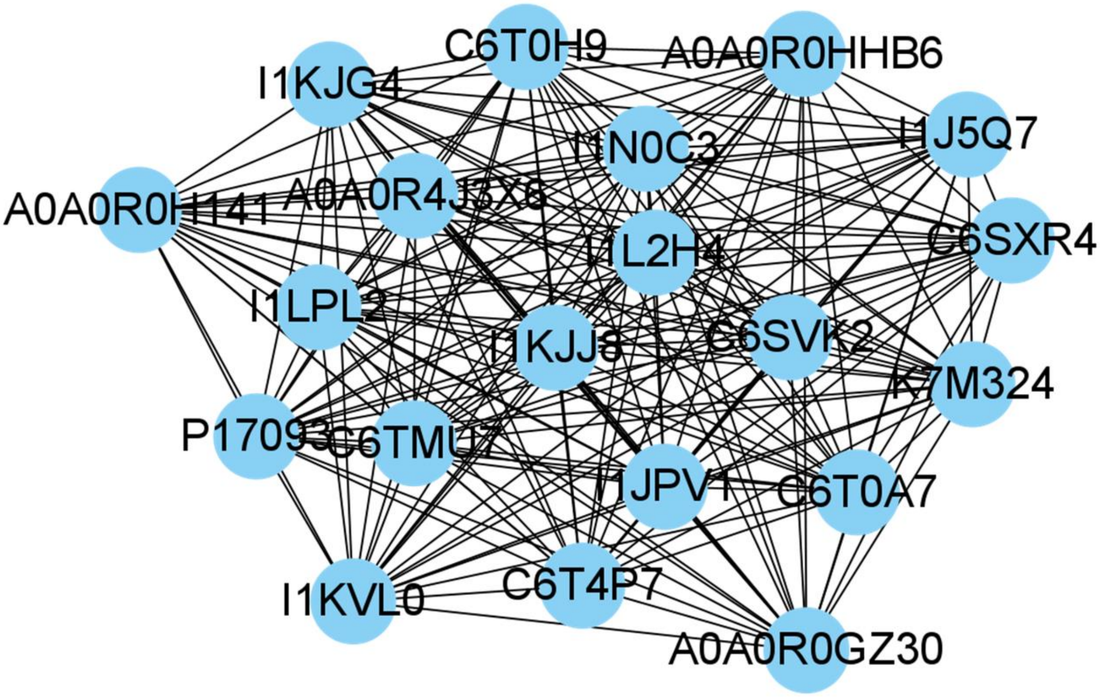

Supplemental Figure S1. Top 20 proteins predicted by the Cytohubba plugin in HN44.

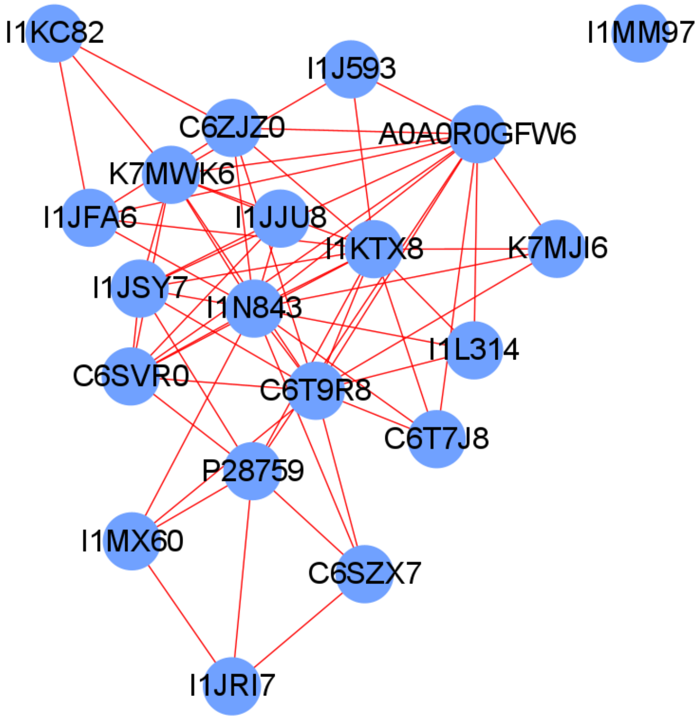

Supplemental Figure S2. Top 20 proteins predicted by the Cytohubba plugin in HN65.
