# Supplementary material for: Mechanism of Mepiquat Chloride Regulating Soybean Response to Drought Stress Revealed by Proteomics
Source: Plants (Basel). 2023 May 19;12(10):2037. doi: 10.3390/plants12102037 (PMC10222127; doi:10.3390/plants12102037)
Supplement: Supplementary file 1 [file plants-12-02037-s001.zip › Supplementary Figures S3 and S4.pdf]

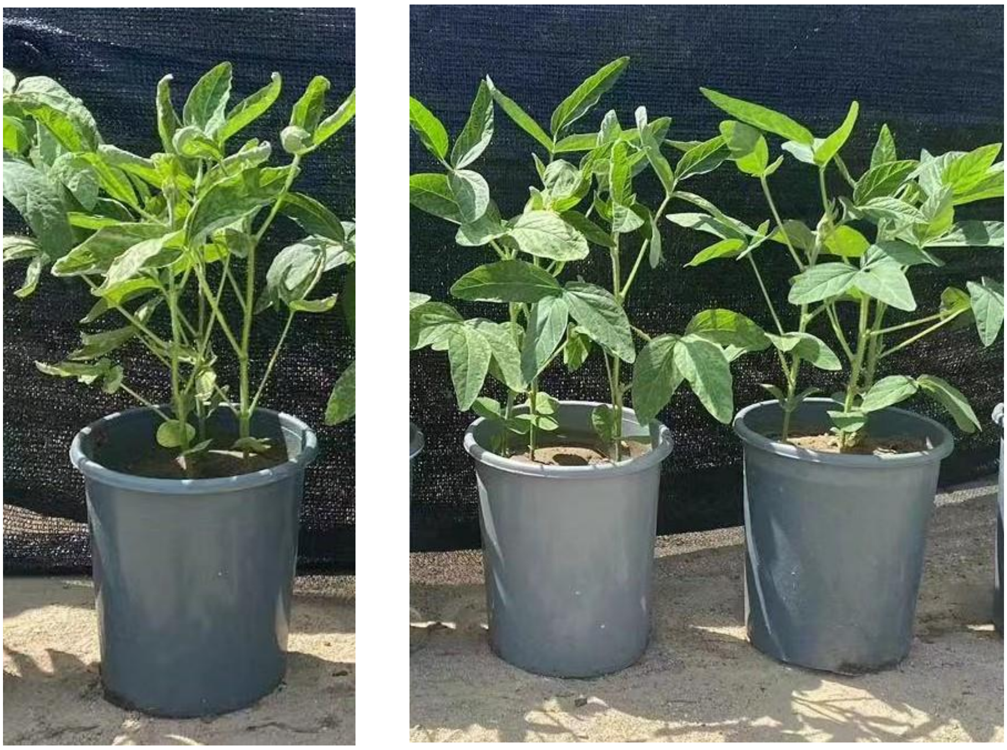

Supplemental Figure S3. The photos of HN65. (left) drought treatment. (Right) MC treatment.

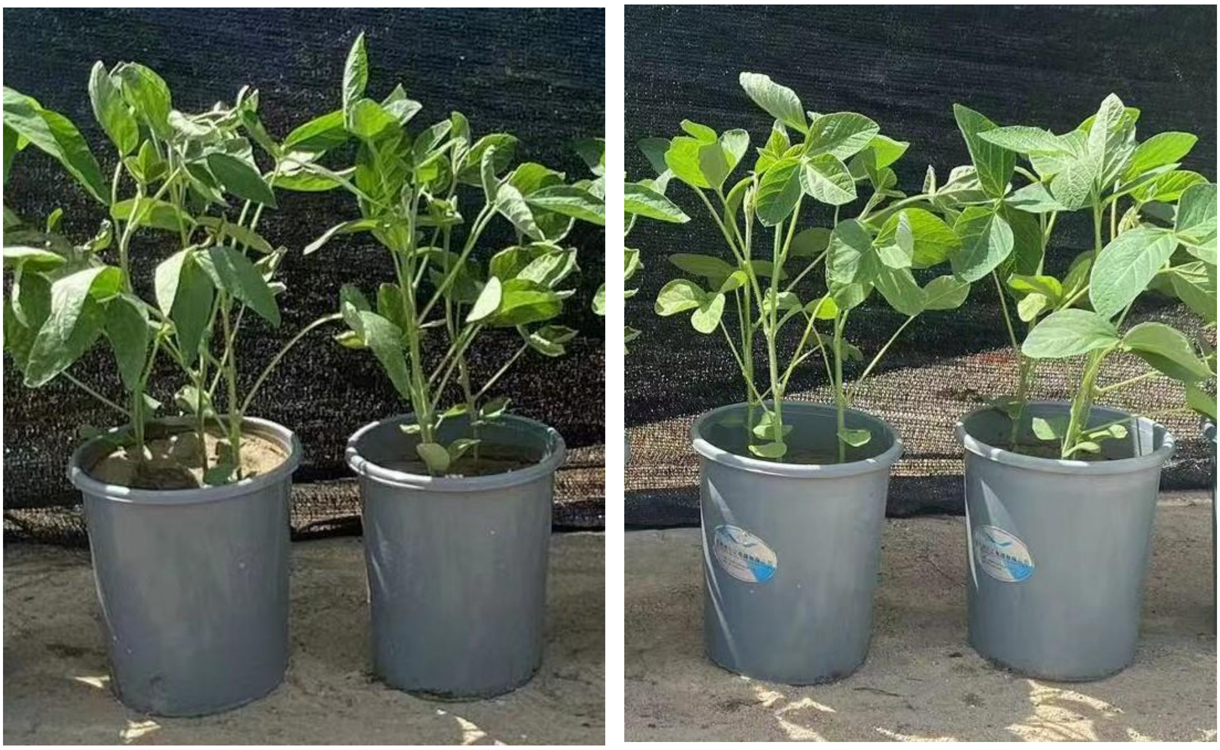

Supplemental Figure S4. The photos of HN44. (left) drought treatment. (Right) MC treatment.
